# Synanthropic rodents as possible reservoirs of shigatoxigenic *Escherichia coli* strains

**DOI:** 10.3389/fcimb.2012.00134

**Published:** 2012-11-01

**Authors:** Ximena Blanco Crivelli, María V. Rumi, Julio C. Carfagnini, Osvaldo Degregorio, Adriana B. Bentancor

**Affiliations:** ^1^Facultad de Ciencias Veterinarias, Cátedra de Patología, Universidad de Buenos AiresBuenos Aires, Argentina; ^2^Facultad de Ciencias Veterinarias, Cátedra de Microbiología, Universidad de Buenos AiresBuenos Aires, Argentina; ^3^Facultad de Ciencias Veterinarias, Cátedra de Salud Pública, Universidad de Buenos AiresBuenos Aires, Argentina

**Keywords:** STEC, hemolytic uremic syndrome, *Rattus*, reservoir, synanthropic

## Abstract

Shigatoxigenic *Escherichia coli* (STEC) strains are worldwide zoonotic pathogen responsible for different cases of human disease including hemolytic uremic syndrome (HUS). Transmission of STEC to humans occurs through the consumption of food and water contaminated by faeces of carriers and by person-to-person contact. The objective of this study was two-fold: (1) to investigate whether synanthropic rodents are possible reservoirs of STEC in the urban area and (2) whether a particular genus out of synanthropic rodent is the principal carrier of STEC. One hundred and forty-five rodents were captured in Buenos Aires City. Screening for *stx*1/*stx*2 and *rfb*O157 was done by PCR from the confluence zone. STEC isolates were further characterized with biochemical tests by standard methods. Additional virulence factors (*eae*, *ehx*A, and *saa*) were also determined by PCR. Forty-one of the rodents were necropsied and sample of kidney and small and large intestine were taken for histopathological diagnosis. The samples sections were stained with hematoxylin-eosin, and observed by light microscopy to evaluate the systemic involvement of these species in natural infections. STEC was isolated from seven out of 27 suspect animals at screening. The following genotypes were found in the STEC strains: *stx*1/*stx*2/*ehx*A (1), *stx*2 (4), *stx*2/*ehx*A (1), *stx*2/*ehx*A/*eae* (1). Neither gross nor microscopic lesions compatible with those produced by Shiga toxin were observed in the studied organs of necropsied rodents. The bivariate analysis including the 145 rodent's data showed that the isolation of STEC is associated positively to *Rattus* genus. This synanthropic species may play a role in the transmissibility of the agent thus being a risk to the susceptible population. Their control should be included specifically in actions to dismiss the contamination of food and water by STEC in the urban area, as additional strategies for epidemiological control.

## Introduction

Shigatoxigenic *Escherichia coli* (STEC) strains are a worldwide zoonotic pathogen responsible for different cases of human disease including diarrhea, hemorrhagic colitis, and hemolytic uremic syndrome (HUS) (Karmali, 1989).

Unlike other commensal *E. coli* strains, STEC strains have several virulence genes which permit the evaluation of its pathogenic nature in the laboratory (*stx*1, *stx*2, *eae, ehx*A, *saa*) (Paton and Paton, 1998a, 2002). These strains may be screened by PCR of *stx* genes in cultured bacteriological samples and subsequent isolation of colonies *stx*^+^.

STEC was reported as inhabitant in the intestine of various animal species: cattle, sheep, pigs, goats (Beutin et al., 1993). There are few references of STEC in synanthropic animals (Hancock et al., 1998; Čižek et al., 1999; Nielsen et al., 2004).

The fecal-oral route is recognized as the major route of transmission, associated with raw or processed food, probably contaminated at some point of the production chain (Meichtri et al., 2004). Over years cattle has been involved as the principal reservoir of STEC, however, isolated strains are partially similar to those which have impact on public health (Boerlin et al., 1999; Rosser et al., 2008).

In the present work we investigated STEC in synanthropic rodents of Buenos Aires City, Argentina. The aim of this study was twofold: (1) to investigate whether synanthropic rodents are possible reservoirs of STEC in the urban area and (2) whether a particular genus of synanthropic rodent is a carrier of STEC.

## Materials and methods

### Study design

A non-probabilistic sampling by convenience was carried out in Buenos Aires City. One hundred and forty-five rodents were captured using live traps (Sherman and cage traps). The traps were placed in parks at intervals of five meters, varying in number according to the surface and the characteristics of each site of capture. The traps remained active for four consecutive nights and they were checked daily in the morning.

The inclusion criteria for the sample from the rodent population of Buenos Aires City were “animals from parks which were captured alive.” We studied the population groups that were validated by relevant epidemiological characteristics. The animals were classified according to genus and species. Each rodent was anesthetized to be weighed, measured, sexed, and their reproductive status was recorded. They were also sampled by two rectal swabs.

The study was approved by the Institutional Animal Care and Use of Experimental Animals, CICUAL, Faculty of Veterinary Science, University of Buenos Aires.

### Detection of *stx*^+^ and isolation of STEC strains

The samples were examined according to standard procedures for isolation of O157:H7 STEC and non-O157 STEC previously described (Bentancor et al., 2007). Briefly, for O157 STEC detection the samples were inoculated in 5 ml of cefixime-tellurite (CT) tripteine soy broth (TBS) for enrichment and incubated 18 h at 37°C. The cultures were streaked onto Sorbitol MacConkey Agar (CT-SMAC) and incubated overnight. Detection of non-O157 was performed inoculating the samples in 5 ml of TSB and incubated overnight at 37°C. The cultures were streaked onto MacConkey Agar (MAC) and incubated overnight.

Screening for *stx*1/*stx*2 and *rfb*O157 from the confluence area (CT-SMAC and MAC) was done by Multiplex PCR (Leotta et al., 2005) using the primers of Pollard et al. (1990) and of Paton and Paton (1998b) (Table 1). To obtain the templates for PCR a sample from the confluence area was taken and suspended in 200 μl of sterile distilled water. The suspension was boiled 10 min in a water bath and centrifuged at 1300 rpm for 5 min. The supernatant was used as the DNA extract. The PCR mixture for each sample had a final volume of 50 μl, and contained 1.5 mM of the two *stx*1 specific primers, 0.3 mM of the two *stx*2 and *rfb*O157 specific primers, 200 mM of each deoxyribonucleoside triphosphate and 1 U of Go Taq (Promega) DNA polymerase in 1 × buffer according to the manufacturer's instructions, and finally 6 μl of the template from sample. PCR was performed in a Mastercycler Gradient Eppendorf under the following conditions: 5 min at 94°C, followed by 30 cycles of: 45 s at 94°C, 30 s at 56°C, 30 s at 72°C and a final step of 7 min at 72°C, maintaining it at 4°C until analysis. 10 μl of each PCR product obtained were electrophoresed on 2% agarose gels and stained with ethidium bromide.

**Table 1:** **Primers used for PCR analysis**.

**Primer**	**Sequence**	**Amplicon**	**Reference**
*stx* 1 a^*^	GAAGAGTCCGTGGGATTACG	130	Pollard et al., 1990
*stx* 1 b^*^	AGCGATGCAGCTATTAATAA		
*stx* 2 a^*^	TTAACCACACCCCACCGGGCAGT	346	Ziebell et al., 2002
*stx* 2 b^*^	GCTCTGGATGCATCTCTGGT		
rfbO157 f^*^	CGGACATCCATGTGATATGG	259	Paton and Paton, 1998a
rfbO157 r^*^	TTGCCTATGTACAGCTAATCC		
*ehxAf*^†^	GCATCATCAAGCGTACGTTCC	534	Paton and Paton, 1998a
*ehxAr*^†^	AATGAGCCAAGCTGGTTAAGCT		
*eaeAf*^†^	GACCCGGCACAAGCATAAGC	384	Paton and Paton, 1998a
*eaeAr*^†^	CCACCTGCAGCAACAAGAGG		
*stx2f*^†^	GGCACTGTCTGAAACTGCTCC	255	Paton and Paton, 1998a
*stx2r*^†^	TCGCCAGTTATCTGACATTCTG		
*stx1f*^†^	ATAAATCGCCATTCGTTGACTAC	180	Paton and Paton, 1998a
*stx1r*^†^	AGAACGCCCACTGAGATCATC		
*saa f*^†^	CGTGATGAACAGGCTATTGC	119	Paton and Paton, 2002
*saa r*^†^	ATGGACATGCCTGTGGCAAC		

* Primers used at PCR screening.

† Primers used in the study of virulence factors.

Positive samples at screening from the confluence zone were considered suspect. Each suspect sample was evaluated for the presence of *stx*^+^ strains up to 50 CFU.

According to the results obtained by PCR, animals were classified into positive (positive screening with isolation of a *stx*^+^ strain), suspect (positive screening without isolation) and negative (pathogen was not detected at screening).

### Biochemical characterization and identification of virulence factors

STEC isolates were further characterized as *E. coli* after gram staining, oxidase, catalase, glucose oxidation-fermentation, indole, methyl red (MR), Voges Proskauer (VP), Simmons citrate, fermentation of sugars (lactose, sucrose, cellobiose, sorbitol, raffinose, dulcitol, rhamnose) H_2_S production, yellow pigment, β-glucuronidase activity, lysine decarboxylase, ornithine decarboxylase, and mobility assessment (Mac Faddin, 2003). RapidID ONE System 20 test (Remel) was also done, and additional virulence factors (*saa, eae, ehx*A) were determined by PCR (Paton and Paton, 1998a, 2002).

### Histopathology

Necropsy of captured animals was performed to determine if STEC carriers had any injury. We looked for gross changes. Kidney, small, and large intestine were removed for histopathological diagnosis. The samples were fixed for 48 h in 10% buffered formalin and processed routinely. Sections of paraffined-embedded tissue were stained with hematoxylin-eosin and evaluated by light microscopy.

### Statistical analysis

The data obtained from each rodent were analyzed with EpiInfo 3.2 (CDC-WHO). We performed bivariate analysis (Chi-squared) and estimated OR and 95% confidence interval for significant variables.

## Results

Rodents captured belonged to the following species: *Deltamys kempi* (*n*:4), *Mus musculus* (*n*:66), *Oligoryzomys flavescens* (*n*:16), *Rattus norvegicus* (*n*:31), *Rattus rattus* (*n*:28).

PCR analysis was performed for all the rodents captured (145 animals). Twenty-seven out of 145 animals were suspected at PCR screening from the confluence area: *Deltamys kempi* (0/4); *Mus musculus* (10/66); *Oligoryzomys flavescens* (1/16); *Rattus norvegicus* (6/31); *Rattus rattus* (10/28) (Table 2).

**Table 2:** **Rodents evaluated as carrier of STEC and virulence profile of isolated strains**.

**Rodent evaluated**	***N*°**	**STEC suspicious carrier at screening**	**Isolation of *stx*^+^ strain**	***Escherichia coli* bacteriological identification**	**O157 serogroup**	**Genotype**				
						***stx*1**	***stx*2**	***eae***	***ehxA***	***saa***
*Rattus norvegicus*	31	6	2	2	0	0	2	0	0	0
*Rattus rattus*	28	10	4	4	0	1	4	1	3	0
*Mus musculus*	66	10	1	1	0	0	1	0	0	0
*Deltamis kempi*	4	0	NA	NA	NA	NA	NA	NA	NA	NA
*Oligoryzom ys flavescens*	16	1	0	NA	NA	NA	NA	NA	NA	NA

NA, not applicable.

Isolation of *stx*^+^ strains was obtained in seven cases out of 27 suspect samples: *Mus musculus* (1/66), *Rattus norvergicus* (2/31), and *Rattus rattus* (4/28). Each isolated strain was identified as *Escherichia coli* by conventional bacteriological test and by RapidID ONE System 20 test. All the isolates were detected as non-O157 strains by PCR.

The following genotypes were found in the STEC strains: *stx*1/*stx*2/*ehx*A (1), *stx*2 (4), *stx*2/*ehx*A (1), *stx*2/*ehx*A/*eae* (1).

Forty-one out of 145 animals evaluated (five positive, four suspect and 32 negative) were necropsied. Neither gross nor microscopic lesions compatible with those produced by Shiga toxin were not observed in the studied organs.

The bivariate analysis including the 145 rodent data showed that the isolation of STEC is associated positively to *Rattus* genus (*p*: 0.01). The possibility of isolating STEC from rodents is higher in *Rattus* than in others genus (OR: 10,62, IC 95%: 1,10-218,07).

## Discussion

Very little is known about the occurrence of STEC in synanthropic rodents. This study differs from others studies which captured rodents and searched for O157 STEC in sampling methods and procedures for isolation of strains (Hancock et al., 1998; Čižek et al., 1999), in our case the presence of O157 was not detected in these samples.

We isolated STEC strains in seven out to 27 suspect animals. This low number of isolates could be related to the number of CFU evaluated in positive animals at PCR screening from the confluence zone. We analyzed 50 CFU from each suspect animal to avoid overestimated rodent species. It is possible that a low proportion of STEC in the suspect samples determinate a low recovery. On the other hand, sampling methods could be the cause of the low efficiency of isolation according to the low sample obtained.

Although rodents from different species were suspected at PCR screening, we observed a higher proportion in individuals of the *Rattus* genus. This fact is important because rodents from *Rattus* genus live in urban areas, overlaying their territory with men, feeding on waste and stowed food which can pollute. The growth of this population is related to their availability to food and water. When we discriminated these individuals by species, the study of carriage in *R. norvegicus* was lower than in *R. rattus*, opposed to the findings of different researchers who have not detected *R. rattus* as a carrier of STEC (Čižek et al., 1999; Nielsen et al., 2004). Differences between *R. rattus* and *R. norvegicus* could not be explained.

In Argentina the epidemiology of HUS included sporadic cases, opposite to large outbreaks of food borne diseases described in others countries. Synanthropic species as other sources deserve to be evaluated to mitigate the endemic presentation of the disease.

The capture of these animals was performed in the urban area, mainly in parks. Although the population of rats of Buenos Aires City is not determinate, larger sample with animals that come from other urban areas would be needed to establish whether there are differences between species in the urban cycle.

In the present study, one strain isolated genotype had *stx*1 and all strains carried *stx*2. The *stx*2 is strongly associated with increased risk of HUS (Rivas et al., 2006). However, none of the isolated strains was from the O157 serogroup, which is most frequent as HUS producer.

The animals captured had no signs of disease; neither gross changes nor microscopic lesions compatible with those produced by Shiga toxin were observed in the studied organs. At the present time, lesions in rodents have been described in animal models which were inoculated orally or gastrointestinally with STEC, or by injection with Stx alone or with lipopolysaccharide component on the outer membrane of this bacterium (Obata, 2010). The absence of lesions may be due to Stx production, amount of Stx receptor (globotriaosylceramide, Gb3) in host cells and their sensitivity to Stx or complex biochemical aspects of the Gb3 membrane microenvironment (Obrig, 2010). Anyway, healthy synanthropic rodents could serve as a reservoir of STEC as occurs in other animal species (Beutin et al., 1993). On the other hand the possibility that the animals consumed contaminated food and could be colonized transiently by STEC could not be excluded. It is difficult to estimate the time of excretion of STEC in wild animals that do not survive in captivity to determine if their carrier status is due to a transient colonization or whether we are in presence of a new reservoir of STEC. In order to obtain a finally conclusion about their role as reservoirs, larger sample would be need.

The results of the present study represent the first finding of STEC in *R. rattus* and show STEC no-O157 is circulating in different synanthropic rodent species of the urban area.

### Conflict of interest statement

The authors declare that the research was conducted in the absence of any commercial or financial relationships that could be construed as a potential conflict of interest.
